# An interactive database of *Leishmania* species distribution in the Americas

**DOI:** 10.1038/s41597-020-0451-5

**Published:** 2020-04-03

**Authors:** Giovanny Herrera, Natalia Barragán, Nicolás Luna, David Martínez, Frasella De Martino, Julián Medina, Sergio Niño, Luisa Páez, Angie Ramírez, Laura Vega, Valeria Velandia, Michelle Vera, María Fernanda Zúñiga, Marius Jean Bottin, Juan David Ramírez

**Affiliations:** 1 Grupo de Investigaciones Microbiológicas – UR (GIMUR), Departamento de Biología, Facultad de Ciencias Naturales, Universidad del Rosario, Bogotá, Colombia; 2 Grupo de Ecología Funcional y Ecosistémica, Universidad del Rosario, Bogotá, Colombia

**Keywords:** Parasitic infection, Parasitology, Research management

## Abstract

The Americas have an elevated number of leishmaniasis cases (accounting for two-thirds of the worldwide disease burden) and circulating *Leishmania* species, and are therefore of interest in terms of epidemiological surveillance. Here, we present a systematic review of *Leishmania* parasite species circulating in the countries of the American continent, together with complementary information on epidemiology and geospatial distribution. A database was built from data published between 1980 and 2018 on *Leishmania* species identified in most of the American countries. A total of 1499 georeferenced points were extracted from published articles and subsequently located to 14 countries in the Americas. This database could be used as a reference when surveilling the occurrence of *Leishmania* species in the continent.

## Abstract (summary)

**Table:** 

Measurement(s)	Distribution
Technology Type(s)	digital curation
Factor Type(s)	reference • political division • type of sample • species • method of identification • geographic coordinates
Sample Characteristic - Organism	Leishmania
Sample Characteristic - Location	North America • South America

Machine-accessible metadata file describing the reported data: 10.6084/m9.figshare.11993970

## Background & Summary

Flagellated parasites of the genus *Leishmania* cause Leishmaniasis, a group of parasitic diseases that are transmitted to humans and other vertebrates through the bite of dipteran sandflies^1,2^. This group of diseases is a global public health problem and is one of the most neglected tropical diseases, with a presence in 98 countries of the world, over 1.3 million new cases every year, and an estimated 350 million people at risk of infection^3^. In the Americas, the disease is present in 19 countries, with Brazil being the most affected country accounting for 96% of all reports^4^. Transmission of the disease is considered stable in countries such as Colombia and Venezuela; whereas, countries such as Argentina, Brazil, and Paraguay have a transmission pattern that shows expansion with new territories affected by the disease^3,4^. There are three different clinical forms of the disease: cutaneous leishmaniasis (CL), mucosal leishmaniasis (ML), and visceral leishmaniasis (VL), with VL showing high mortality rates in patients due to the absence of rapid diagnostic tests or timely treatment^5^. In total, 75% of the CL cases occur in countries of the Americas highlighting the epidemiological relevance of the continent^6^.

There are around 53 different species of the parasite, 20 of which have been found in humans^7^. This great diversity of species is relevant as some have been associated with the clinical form of the disease and resistance to conventional treatments^8–10^. In this sense, the American continent represents a special scenario for the disease not only due to its high disease burden, but also for the high concentration of different species in the same country, reaching up to ten different species within the same territory^11,12^.

Despite the relevance of species identification in clinical and epidemiological terms, this is not carried out routinely in most clinical centers and is generally limited to research laboratories^13,14^. Based on existing information, some countries have attempted to describe the circulating species in their territories^11,15–17^, without reaching the micro-geographical scale. Although many of the publications include the geographic coordinates of sample collection as well as other elements that are important for epidemiological surveillance, much of this information is not currently included in the descriptions recorded by each country. Further, there is no centralized information that accounts for the situation at the continental level, which could contribute to surveillance of the disease^4^.

**Fig. 1:**
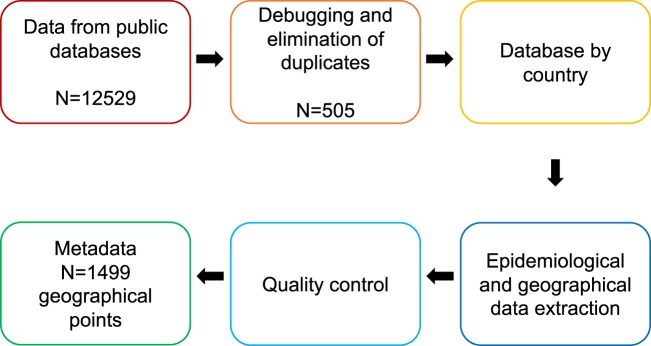
Data compilation and debugging process.

Therefore, the objective of this work was to generate a public database with updated and georeferenced information on *Leishmania* species present in countries of the American continent. The information included is the product of a systematic review of articles extracted from three different databases (Fig. 1). This information improves our knowledge on the occurrence of parasite species in the continent, allowing for the design of better public health surveillance strategies that reduce the incidence of the disease.

## Methods

### A systematic review of the literature

To construct this metadata, two different researchers independently carried out the search and selection of the articles for each of the 32 countries. If there was a discrepancy between the results, a third investigator resolved the conflict. The data were not disclosed among the researchers until the final consolidation was obtained for each of the parties. To determine the usability of the identified article, the description of some species’ identification methods in the title or summary (first debug) was defined as an inclusion criterion. Subsequently, an independent group of two researchers, who carried out the search, proceeded to perform a second evaluation of the article, considering the complete information described in the methods and results, and define its final inclusion or exclusion (second debug). Similarly, this group began with the extraction of the data included in the metadata, and a third group reviewed the coordinates in detail and normalized the names (third debug).

### Inclusion and exclusion criteria

For this study, we considered those articles with clinical (method of identification, sample type, and species identified) and geographical complete information. All languages were considered (Spanish, English, and Portuguese). Information was searched for in the abstract and full article. We excluded some articles without the full (.pdf) version or with incomplete information. Some articles that used techniques that did not correctly identify the species were also excluded. For some descriptions, geographical coordinates were input using the name of the territory where the sample was collected.

### Description of the database fields

The information available on *Leishmania* species in the Americas was collected and systematized into 15 fields of *Leishmania* in the Americas database. In turn, the database’s 15 fields were considered within six categories: (a) Reference (DB, title, author, year, and journal), (b) Political division (country, state, and municipality), (c) Type of sample (sample and source), (d) Species (species, scientific name, genus; includes the *Leishmania* species and the host in which it was found), (e) Method of identification of the *Leishmania* species (method) and (f) Geographical coordinates (latitude and longitude). Some of the categories mentioned above will be detailed below:

#### Political division

This refers to the detailed description of the locality in which the *Leishmania* species were reported.

#### Type of sample

The sample field refers to the type of sample used to identify *Leishmania* species. The following types of samples were considered: (a) Bone marrow/liver/spleen, (b) Skin, (c) Tissue, (d) Serum, and (e) Insect, which generally refers to the vectors of *Leishmania* parasites. It is important to mention that the first four types of samples apply only to mammals. The database also contains a field with the sample’s source, which refers to the sampling method or primary sample the author used to obtain the final sample employed to identify *Leishmania* species. In this field, the following sources were considered: (a) Aspirate, (b) Biopsy, (c) Dermal scrapping, (d) Blood, and (e) Insect.

#### Identification method

Only studies in which molecular and/or serological tests were performed are included in this database. Thus, the following tests are included within this field: (a) Immunoassay (which includes tests such as Enzyme-Linked ImmunoSorbent Assay (ELISA), Indirect Immunofluorescence (IIF), monoclonal antibodies), (b) Polymerase Chain Reaction (PCR), and (c) Multilocus Enzyme Electrophoresis (MLEE).

#### Species

The *Leishmania* species identified in the systematic review are detailed in the species field, and the scientific name and genus fields refer to the mammal or arthropod in which the parasite was found.

### Information about the databases used as sources

The database was constructed based on scientific articles published in the EMBASE, PubMed, and LILACS repositories. To search for scientific articles, the researchers did not establish a certain period and instead, used the following search algorithm for each country of the Americas: “Leishmania AND country name”. Articles were excluded if a species identification was not carried out, or their objective was a systematic review or a clinical trial. Similarly, only freely available items were considered.

Once the articles were read and refined according to the criteria mentioned above, a database by country was constructed. Subsequently, the articles already refined by country were collected into a database that constitutes the metadata of the project. Additionally, three independent rounds of depuration of the data, extracted from the articles, were carried out to avoid including articles that did not conform to the required parameters and to verify the geographical location of the data collected in the different studies. Finally, the database fields were standardized so that the identification methods, *Leishmania* species, type of sample, and coordinates were in the same format and under the same name.

### Georeferencing process

From the selected articles, the coordinates were taken and assigned to the reported *Leishmania* species. The geographical coordinates (latitude and longitude) presented in this database are in decimal format. In those cases in which the articles specified the coordinates in a different format, the coordinates were converted into decimal format using a web page of geographical coordinates (https://www.gps-coordinates.net/). If the article specified the site name where the species was reported, this name was entered into the web page mentioned above and the decimal coordinates were extracted. All of the coordinates reported in the articles were double-checked to detect errors in the allocation of the geographical location of the species found. The coordinate system used for all geographic records was WGS84.

## Data Records

The workflow used for the construction of the database is detailed in Fig. 1. A total of 505 articles with 1499 records of *Leishmania* species were identified in 14 countries of the American continent. This database is stored at Harvard Dataverse^18^. Each of the entries corresponds to the geographic and epidemiological information of a *Leishmania* species, as described above. The metadata can be visualized as an interactive map on http://worldmap.harvard.edu/data/geonode:leishmania_fy, and the metadata file is available to be downloaded as a tab file on 10.34848/FK2_leshmania_ds.

**Fig. 2:**
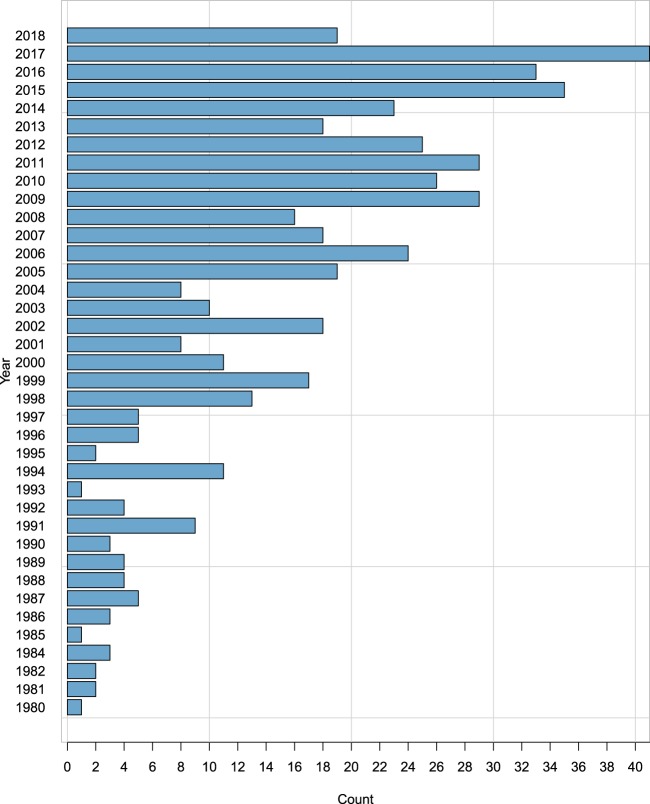
Description of the number of articles reported per year.

**Fig. 3:**
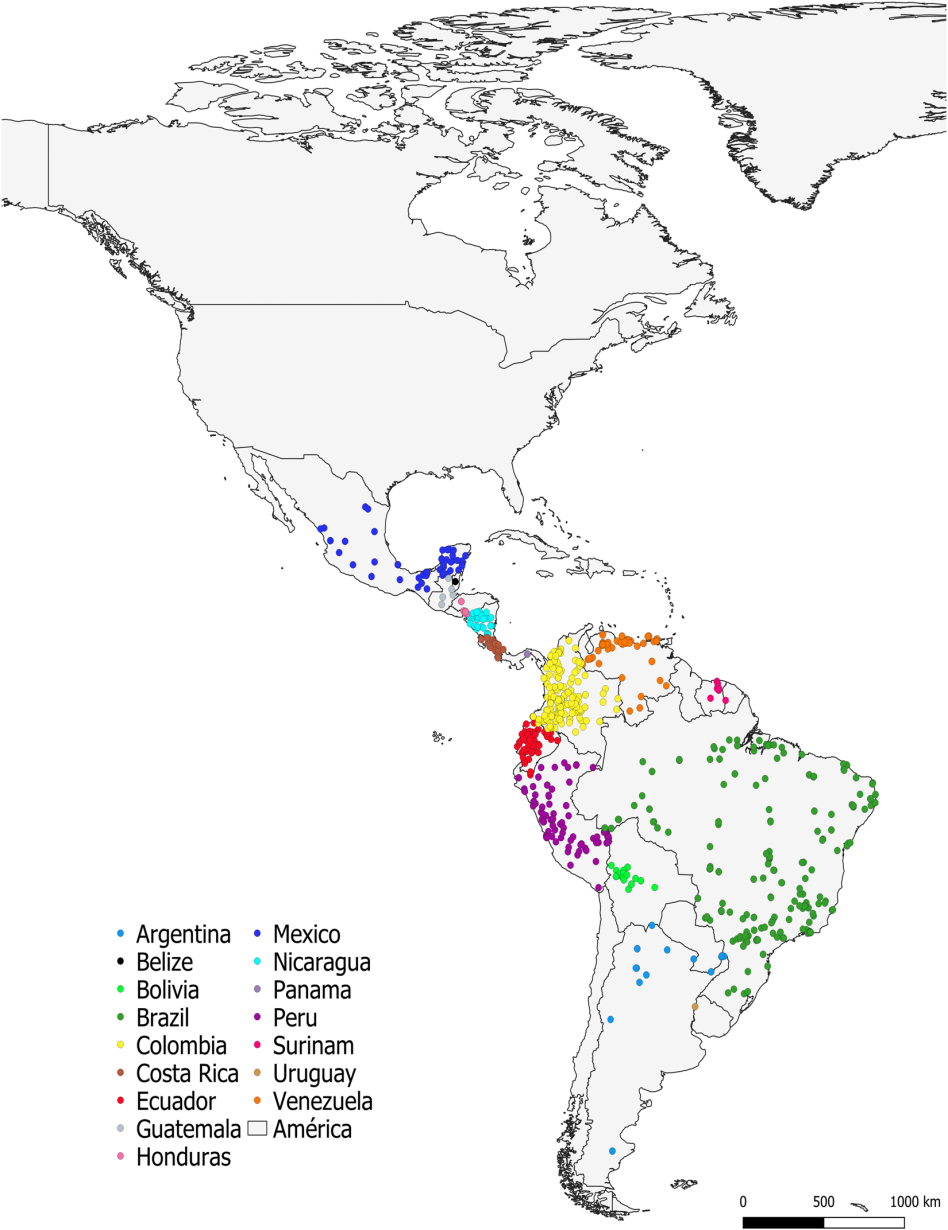
Geographic distribution of the occurrence of *Leishmania* reports by country.

The information contained in this database corresponds to records published between 1980 and 2018, with the years 1994, 2012, 2016, and 2017 containing the greatest number of reports (Fig. 2), covering a wide geographical range that includes countries from Mexico to Argentina. More than 60% of the reports originated from Brazil, Colombia, and Peru (Fig. 3).

**Fig. 4:**
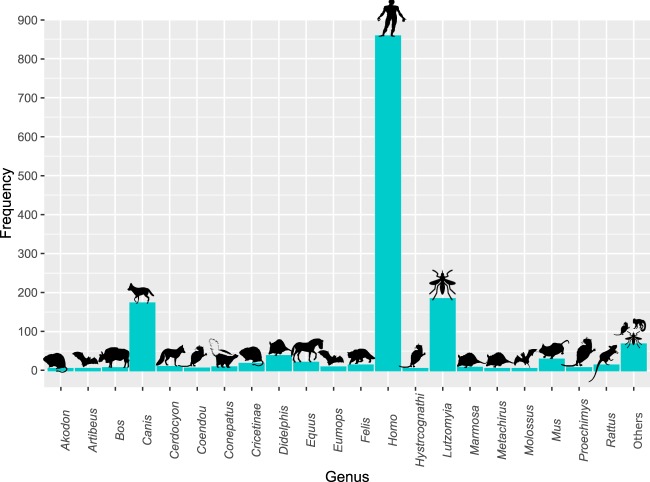
Frequency of reports by host.

Brazil showed the largest number of species (15 in total), followed by Ecuador and Peru (11 species each) (Table 1). In total, 20 species were found circulating in the Americas, in a wide range of hosts (26 in total). In total, 57% of the reports were based on human samples, followed by samples from insects and canines (Fig. 4, Supplementary Table 1). Tissue was the most widely used type of sample, followed by serum and insects, and PCR was the most common technique used.

**Table 1:** Summary of the clinical and epidemiological information by country.

Country	N of species	N of records	N of records obtained from Biopsy	N of records obtained from Dermal	N of records obtained from Blood	N of records obtained from Insect	N of records obtained from Aspirate
Argentina	7	38	7	15	3	7	6
Belice	2	7	7	—	—	—	—
Bolivia	6	58	22	1	3	11	21
Brazil	15	470	229	13	159	60	9
Colombia	10	264	105	105	29	24	1
Costa Rica	3	20	1	—	—	—	19
Ecuador	11	99	59	12	—	28	—
Guatemala	2	5	2	—	—	3	—
Honduras	2	10	3	—	—	—	7
Mexico	9	138	100	3	12	5	18
Nicaragua	4	51	17	6	—	—	28
Panamá	4	6	2	1	—	—	3
Peru	11	236	145	39	3	28	21
Suriname	5	14	9	—	—	5	—
Uruguay	1	1	1	—	—	—	—
Venezuela	8	81	45	9	8	16	3
Total		1502	756	204	217	189	136

## Technical Validation

Initially, the debugging process previously described allowed for the correct selection of data to be included. This step assured the reliability of the information included in the database. To reduce typographical errors, normalization of the technique’s names used for the identification of species was carried out by categorizing them and reducing detailed information such as the types of probes and primers used. Likewise, the host and sample type data were standardized to uniformly determine the provenance of the sample. In the cases in which the sample collection coordinate was not specified, researchers used a gelocation web page (https://www.gps-coordinates.net/) to search for the name reported in the scientific article. The last verification was made using a Shiny app code to avoid errors in the geographical location of each point with real-time visualization of the position of all records (see code availability).

Finally, the names of the species described in each of the articles were written without considering the subgenus to standardize and avoid typographical errors according to the classification of Akhoundi *et al*.^7^.

Despite the limitations of serological methods to identify *Leishmania* species (monoclonal antibodies identify some species complexes, but such methods are not highly accurate for identification at the species level), we decided to include these metadata because of their epidemiological importance, and at the same time, we created a new database version without the cases identified by those methods. Ramirez *et al*. in 2014 demonstrated a high concordance among serological tests, MLEE, and PCR, showing the importance of this type of data^19^. Both datasets are available at the previous dataverse link^18^.

## Usage Notes

Considering the high volume of data retrieved for some countries such as Brazil, Colombia, Peru, and Mexico, this database may contain some errors specific to this type of systematic review. To facilitate data management, a normalization process was carried out, avoiding the characteristics of some techniques used in the identification of parasites, grouping them into larger categories. It is necessary to consider that some coordinates were assigned considering only the name of the place described in the scientific articles, and their precise locations may vary.

Despite the limitations highlighted above, this database is a useful tool in epidemiological surveillance for all of the countries in the region, especially in contrast to the descriptions of vectors and reservoirs made throughout the continent. It is worth noting that this is the first database related to leishmaniasis that is offered to the community to carry out studies that promote better surveillance and control measures of the disease. This database is a powerful tool for the public health surveillance systems of each country and decision-makers because it allows for visualization, in a dynamic way, of the distribution of *Leishmania* species in each country but also on the whole continent. This visualization may be particularly useful for countries with shared borders when trying to devise strategies to decrease the disease burden. Finally, we encourage the governmental authorities and the Pan-American Health Organization (PAHO) to concentrate their reports and contribute to future updates of this database.

## Supplementary information

Supplementary information

## Data Availability

A GitHub record of this project is accessible at https://github.com/gimur/leishmaniadb. It includes the Shiny app code used to verify the coordinates of each report.
